# Rye Bread with an Admixture of Malt as Food for Horses

**Published:** 1896-09

**Authors:** 


RYE-BREAD WITH AN ADMIXTURE OF MALT AS FOOD FOR HORSES. The idea of enriching the bread for horses in fatty principles, and especially in nitrogen, is not new; bran, gluten, peanuts, beans, peas, and quantities of other vegetable and even animal products have been recommended for this purpose. Lately, a baker in Flanders has proposed to add the brewery malt, previously dried, by putting two parts of dried malt to three parts of rye-meal; he finds that the richness of bread in albuminous and fatty materials is almost doubled. This makes very valuable rations for the work-horse as well as for fattening cattle.—*Annales de Médecine Vétérinaire*, March, 1896.

